# Supplementary material for: Hierarchical Bayesian Modelling Improves Microstructural Parameter Mapping in Diffusion and Exchange MRI Data
Source: NMR Biomed. 2026 May 11;39:e70277. doi: 10.1002/nbm.70277 (PMC13160881; doi:10.1002/nbm.70277)
Supplement: Supplementary file 1 — Supporting Information [file NBM-39-e70277-s001.pdf]

SUPPORTING FIGURES AND TABLES

S1 | COMPARISON BETWEEN HBM AND SMOOTHING (SIMULATIONS)

To determine whether simple filtering methods could achieve the same parameter map quality as the HBM approach, the AXR data generated in Section 2.3.2 was smoothed using four filters before fitting the FEXI AXR model using least-squares (LSQ) regression (as in Section 2.4.1): (i) Gaussian filter with kernel size 3 × 3; (ii) Gaussian filter with kernel size 5 × 5; (iii) median filter with kernel size 3 × 3, and; (iv) median filter with kernel size 5 × 5. The derived parameter maps are shown in Figure S1.1A, with corresponding error maps in Figure S1.1B.

The median filters outperformed the Gaussian filters, with larger filter sizes also performing better. While the best-performing filters (Gaussian 5 × 5, median 5 × 5) improved parameter fitting and reduced errors compared to unfiltered LSQ-derived maps, smoothing the source data effectively increased the voxel size; this introduced spatial correlations in the derived parameter maps and reduced contrast between the 'WM' and 'GM' ROIs. The HBM approach improved parameter estimation while preserving spatial resolution and maintaining contrast between the ROIs.

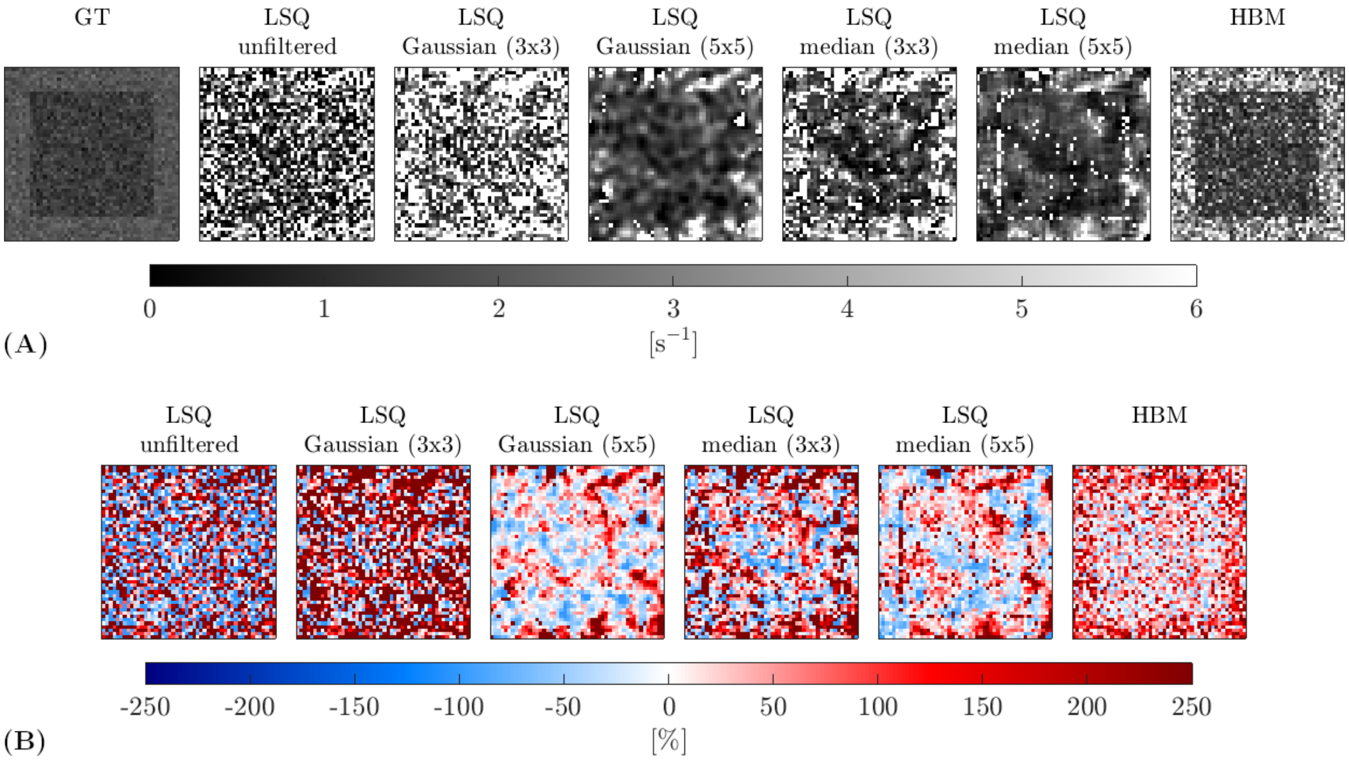

FIGURE S1.1 | **(A)**. Apparent exchange rate (AXR) parameter maps for the simulated data, showing ground truth (GT) values, the unfiltered least-squares (LSQ) output (replicated from Figure 2B), the filtered LSQ-derived outputs (where source data was smoothed using Gaussian and median filters, each with 3 × 3 and 5 × 5 kernels), and the hierarchical Bayesian modelling (HBM) output (replicated from Figure 2B). Filtering introduced spatial correlations, which were avoided using the HBM approach. **(B)**. Errors for the derived AXR parameter maps.

S2 | COMPARISON BETWEEN HBM AND NON-LOCAL MEANS FILTERS (SIMULATIONS)

To determine whether non-local means (NLM) filtering methods could achieve the same parameter map quality as the HBM approach, AXR data was generated as in Section 2.3.2 but with matrix size 25 × 25 × 4 (giving the same number of voxels) to satisfy 3D data requirement of the NLM filter. This data was denoised using a NLM filter (dipy.denoise.nlmeans.nlmeans[55]) before fitting the FEXI AXR model using least-squares (LSQ) regression (as in Section 2.4.1). The derived parameter maps are shown in Figure S2.1A, with corresponding error maps in Figure S2.1B. NLM denoising improved parameter fitting and reduced errors in the LSQ-derived maps; however, NLM was not as effective at stabilising the model fitting compared to the HMB approach.

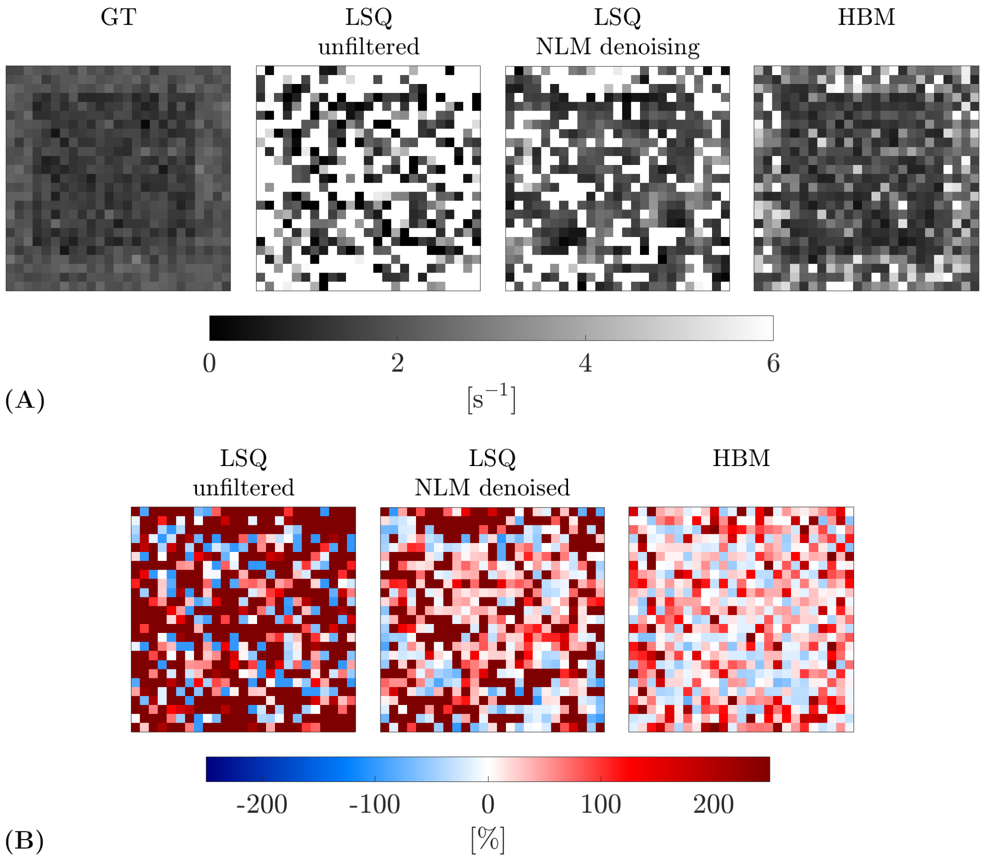

FIGURE S2.1 | **(A)**. Apparent exchange rate (AXR) parameter maps for the simulated data, showing ground truth (GT) values, the unfiltered least-squares (LSQ) output, the non-local-means (NLM) denoised LSQ-derived outputs, and the hierarchical Bayesian modelling (HBM) output. **(B)**. Errors for the derived AXR parameter maps.

S3 | IMPACT OF DIFFERENT REGIONAL PRIORS ON HBM (SIMULATIONS)

Model parameters were generated for two ROIs representing healthy WM and GM as in Section 2.3.2. WMH regions were also simulated, using: $D_{wm} \sim \mathcal{N}(2.00, 0.10)$ µm^2/ms, $\sigma_{wm} \sim \mathcal{N}(0.20, 0.03)$, AXR$_{wm} \sim \mathcal{N}(8.00, 2.00)$ s^{-1}. The ground truth parameter map was generated with the WMH ROI in two distinct spatial locations, and 1133/1136/231 voxels in the WM/GM/WMH ROIs respectively. The WMH ROI size was chosen to be approximately 20% of the WM ROI, in line with the in vivo data. Noisy BBB-FEXI signals were generated as in Section 2.3.2.

The FEXI AXR model was fitted to the signals using the LSQ and HBM approaches. The HBM model was run three times using different prior configurations: (i) one global prior ($k = 1$); (ii) two regional priors ($k = 2$), one each for the WM/GM ROIs with WMH voxels included in the WM prior, and; (iii) three regional priors ($k = 3$), with an additional prior for the WMH ROI.

AXR parameter maps are shown in Figure S3.1; error maps and statistical analyses are provided in Figure S3.2 and Table S3.1 respectively. Figure S3.3A shows AXR distributions and Figure S3.3B the CNR between WM and WMH ROIs. Qualitatively, the HBM outputs using $k = 2$ and $k = 3$ regional priors provided better contrast between all ROIs than when using $k = 1$ global prior, where contrast between WM and GM was reduced. WMH ROIs remained well-resolved for all HBM outputs; however, CNR between WM and WMH was highest when using $k = 3$ regional priors.

These results support the observations in cSVD patients in Figures 6 and 7, where using a separate prior for pathological tissues was found to improve accuracy and contrast. However, it is important to note that there is likely to be some interplay between the chosen number of regional priors (k), the number of samples (voxels) in each prior ROI, and the underlying regional parameter distributions; this should be considered when using different datasets, as it may influence the ability of the HBM method to resolve distinct regions accurately.

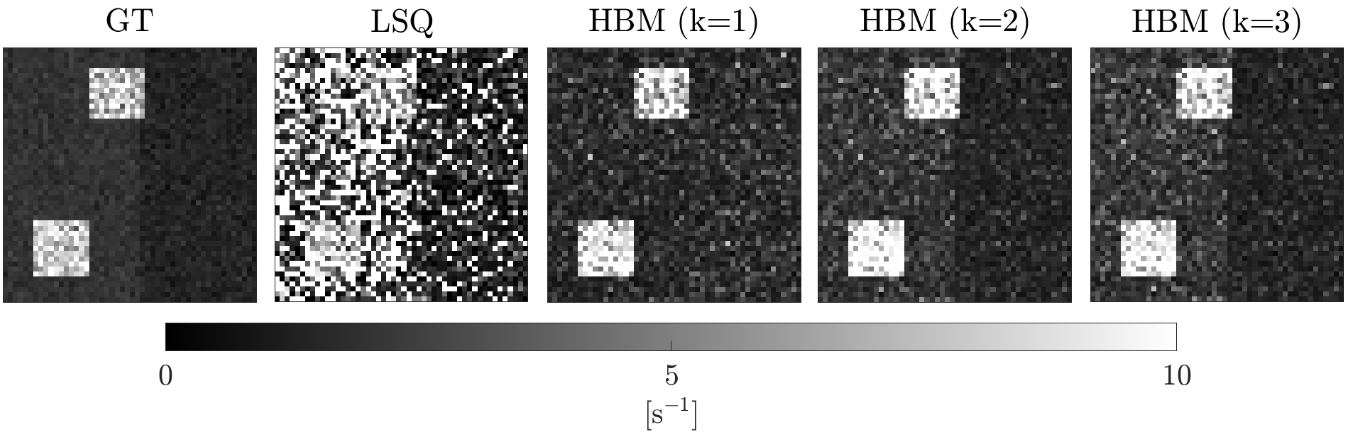

FIGURE S3.1 | Apparent exchange rate (AXR) parameter maps derived using the least-squares (LSQ) and hierarchical Bayesian modelling (HBM) approaches in simulated data; k indicates the number of priors used for the HBM method, where $k = 1$ denotes one global prior, $k = 2$ denotes two priors (WM and GM), and $k = 3$ denotes three priors (WM, GM and WMH). The simulated data shows WM on the left, GM on the right, and two WMH square regions embedded within the WM.

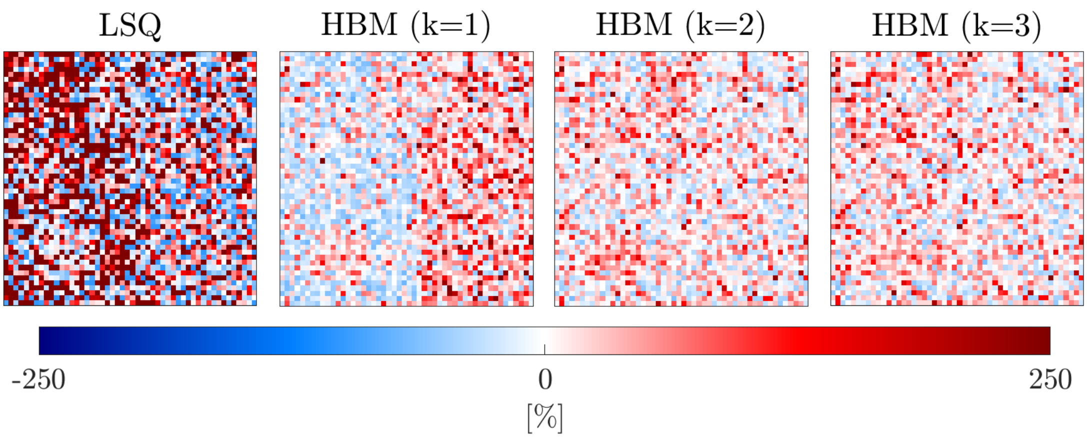

FIGURE S3.2 | Percent errors maps for the least-squares (LSQ) output and hierarchical Bayesian modelling (HBM) outputs in simulated data; k indicates the number of priors used for the HBM method, where $k = 1$ denotes one global prior, $k = 2$ denotes two priors (WM and GM), and $k = 3$ denotes three priors (WM, GM and WMH).

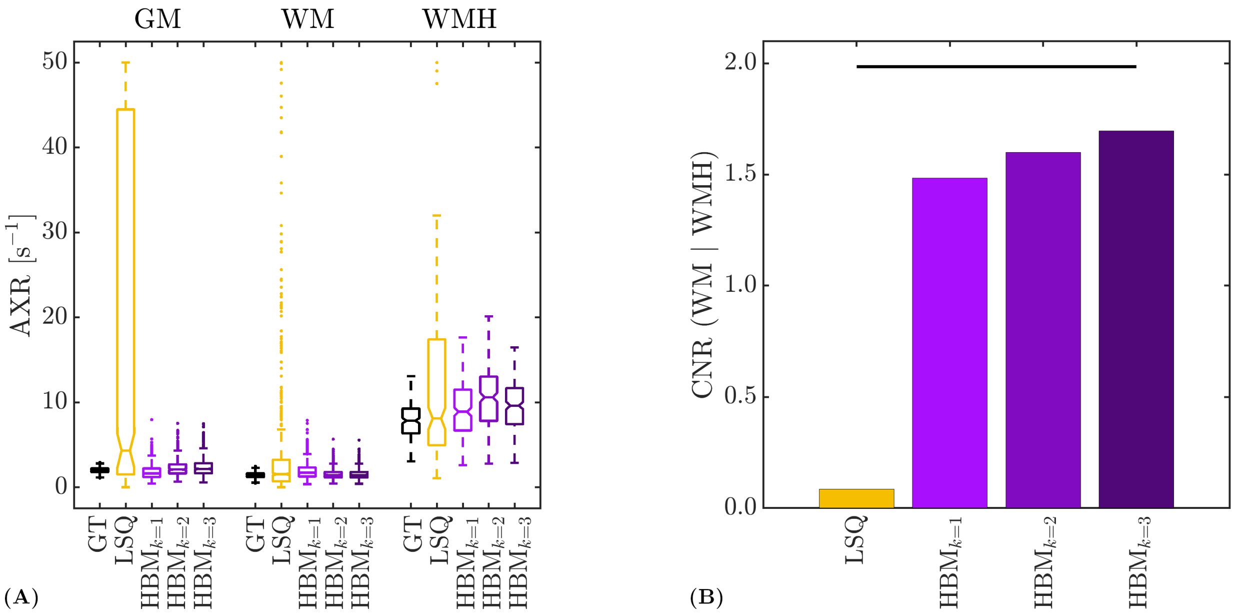

FIGURE S3.3 | **(A)**. Boxplots of voxelwise apparent exchange rate (AXR) estimates are shown for simulated data mimicking grey matter (GM), white matter (WM) and white matter hyperintense (WMH) regions for the least squares (LSQ) and hierarchical Bayesian modelling (HBM) approaches; the number of regional priors used for the HBM approach is indicated by the subscript, where $k = 1$ indicates one global prior, $k = 2$ indicates two priors (WM, GM), and $k = 3$ indicates three priors (WM, GM, WMH). Using a separate prior for WMH (i.e. $k = 3$) improved parameter accuracy, particularly in WM and GM. **(B)**. Contrast-to-noise ratio (CNR) between WM and WMH regions. The CNR was higher for $k = 3$ than for $k = 1, 2$, indicating superior discrimination of WMH AXR alterations when using a separate WMH prior.

TABLE S3.1 | Error metrics for the simulated BBB-FEXI (SNR = 30) data. Extreme fits were defined as parameter values within 1% of the fitting bounds; values in brackets indicate the total proportion of voxels containing one or more parameters with extreme fits.

		AXR model			
		D [μm^2/ms]	σ [a.u.]	AXR	
**RMSE**	LSQ	5.76×10^{-2}	8.79×10^{-2}	20.4	
	HBM (k=1)	3.92×10^{-2}	2.78×10^{-2}	1.4	
	HBM (k=2)	3.56×10^{-2}	2.70×10^{-2}	1.5	
	HBM (k=3)	3.36×10^{-2}	2.74×10^{-2}	1.3	
**Bias**	LSQ	-0.32×10^{-2}	1.99×10^{-2}	9.79	
	HBM (k=1)	0.02×10^{-2}	0.01×10^{-2}	0.27	
	HBM (k=2)	0.02×10^{-2}	0.11×10^{-2}	0.42	
	HBM (k=3)	0.04×10^{-2}	0.07×10^{-2}	0.38	
**CNR** (WM-GM)	GT	2.5	0.8	1.0	
	LSQ	1.7	0.1	0.1	
	HBM (k=1)	1.9	0.6	0.1	
	HBM (k=2)	2.3	0.9	0.5	
	HBM (k=3)	2.4	0.8	0.5	
**CNR** (WM-WMH)	GT	7.2	1.2	2.0	
	LSQ	6.1	0.3	0.1	
	HBM (k=1)	7.2	1.1	1.5	
	HBM (k=2)	7.4	1.1	1.6	
	HBM (k=3)	9.0	1.1	1.7	
**Extreme fits**	LSQ	0.0	6.6	19.5	(25.4)
	HBM (k=1)	0.0	0.0	0.0	(0.0)
	HBM (k=2)	0.0	0.0	0.0	(0.0)
	HBM (k=3)	0.0	0.0	0.0	(0.0)

Abbreviations: AXR, apparent exchange rate; BBB, blood-brain barrier; CNR, contrast-to-noise ratio; DKI, diffusion kurtosis imaging; FEXI, filter exchange imaging; RMSE, root mean squared error; SNR, signal-to-noise ratio.

S4 | CEREBRAL SMALL VESSEL DISEASE PATIENTS

All parameters of the FEXI AXR model are shown here for the two exemplar cSVD subjects (Figures S4.1 and S4.2 respectively), both for the LSQ approach and for the HBM approach with $k = 3$ regional priors (white matter, grey matter, and white matter hyperintensity). Noise was substantially reduced in the HBM parameter maps, revealing an increase in AXR in WMH relative to WM; by comparison, high noise levels in the LSQ maps obscured any alterations in the WMH AXR.

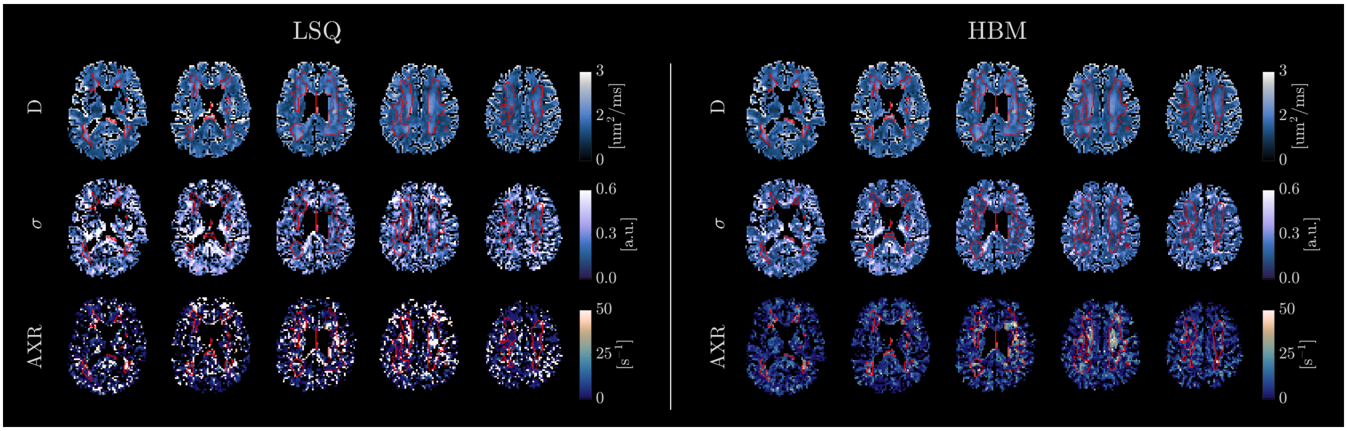

FIGURE S4.1 | Cerebral small vessel disease subject S1. Apparent diffusion coefficient (D), filter exchange (σ) and apparent exchange rate (AXR) parameter maps, derived using least-squares (LSQ) and hierarchical Bayesian modelling (HBM) (with $k = 3$ regional priors) approaches. Noise was substantially reduced in the HBM parameter maps, revealing regions of increased AXR within white matter hyperintensities (outlined in red) that are not visible in the LSQ maps.

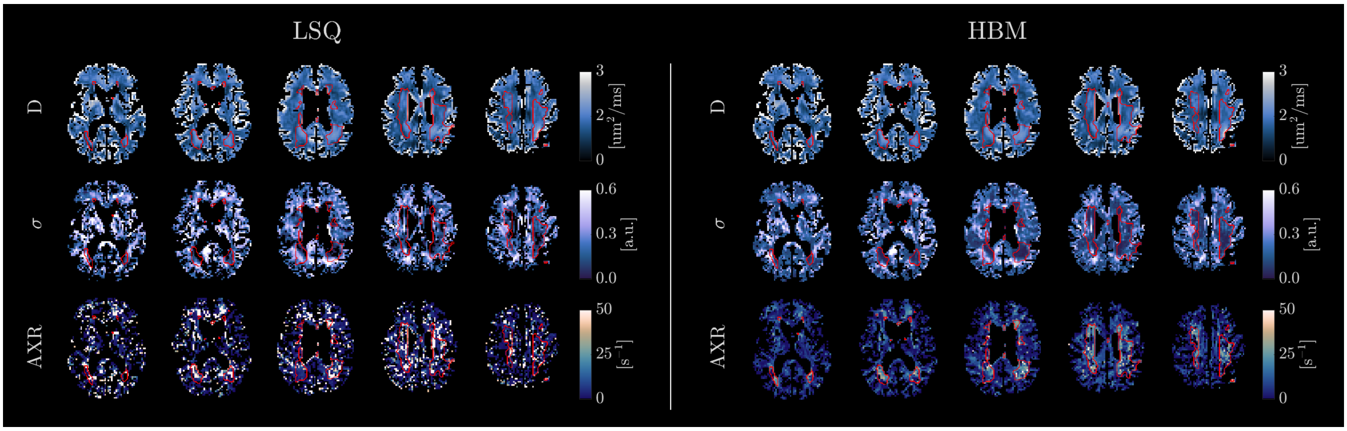

FIGURE S4.2 | Cerebral small vessel disease subject S2. Apparent diffusion coefficient (D), filter exchange (σ) and apparent exchange rate (AXR) parameter maps, derived using least-squares (LSQ) and hierarchical Bayesian modelling (HBM) (with $k = 3$ regional priors) approaches. Noise was substantially reduced in the HBM parameter maps, revealing regions of increased AXR within white matter hyperintensities (outlined in red) that are not visible in the LSQ maps.
